# *Parvimonas micra* promotes carcinogenesis of colorectal cancer through phenyllactic acid‐induced DNA damage

**DOI:** 10.1002/ctm2.70667

**Published:** 2026-05-03

**Authors:** Shuang Guo, Mujia Cao, Jinjie Wu, Wenhao Ma, Dayi Liang, Hongyu Xie, Yanchun Xie, Zhanhao Luo, Peng Lai, Danling Liu, Wanyi Zeng, Jingbiao Zheng, Mengze Xing, Xiqi Yin, Min Xia, Zhen He

**Affiliations:** ^1^ Guangdong Provincial Key Laboratory of Colorectal and Pelvic Floor Diseases The Sixth Affiliated Hospital Sun Yat‐Sen University Guangzhou China; ^2^ School of Public Health Sun Yat‐Sen University Guangzhou China; ^3^ Biomedical Innovation Center The Sixth Affiliated Hospital, Sun Yat‐Sen University Guangzhou China; ^4^ Department of General Surgery (Colorectal Surgery) The Sixth Affiliated Hospital, Sun Yat‐Sen University Guangzhou China; ^5^ Key Laboratory of Human Microbiome and Chronic Diseases (Sun Yat‐Sen University), Ministry of Education Guangzhou China; ^6^ Department of Anesthesia The Sixth Affiliated Hospital, Sun Yat‐Sen University Guangzhou China

**Keywords:** carcinogenesis, colorectal cancer, DNA damage, metabolomics, microbiota

## Abstract

Recent studies have demonstrated the significance of gut microbiota in the colorectal cancer (CRC) pathogenesis. But their role in carcinogenesis remains to be established. Thus, we established a clinical cohort and the faecal samples from CRC and healthy control were collected. Our metagenomic analysis found that the presence of *Parvimonas micra* exhibited the most significant relationship with the occurrence of CRC. Increased colonisation of *P. micra* in CRC was validated with analysis of 1379 faecal metagenomes from eight public cohorts. Untargeted metabolomics subsequently identified an accumulation of phenyllactic acid (PLA) in faecal samples from CRC patients. Higher concentration of PLA was detected in the supernatant from our isolated *P. micra*. Whole‐genome sequencing confirmed that a series of genes associated with PLA biosynthesis such as *pdhD* were observed in the *P. micra* genome. Importantly, both *P. micra* and PLA‐induced carcinogenesis in *Apc* ^Min/+^ and azoxymethane/dextran sulphate sodium salt mice model. The roles of *P. micra* and PLA in CRC development were associated with DNA damage. Engineered *Escherichia coli BL21* that encoded the heterologous *pdhD* from *P. micra* could also induce DNA damage. Mechanically, PLA‐induced DNA damage and CRC carcinogenesis were significantly alleviated in *Ahr^−/−^* mice. Aryl hydrocarbon receptor (AHR) inhibitor exhibited a therapeutic potential to reduce mice carcinogenesis. These findings established the role of *P. micra* and its metabolite, therefore providing diagnostic and therapeutic targets for treating CRC.

## Graphical abstract

Multiomics analysis of the faecal samples from CRC patients found that upregulation of *P. micra* and PLA was associated with the CRC development. PLA from *P. micra* via *pdhD* genes could induce DNA damage via AHR signalling pathway to mediate carcinogenesis.

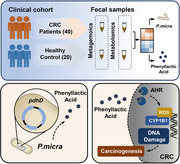

## 1 INTRODUCTION

Colorectal cancer (CRC) is the third most common cancer in the world and the mortality rate has risen to the second highest.^1^ Occurrence of CRC has been modulated by various factors such as external environment, diet, genetic factors, internal immune and gut microbiota.^2^ Recent studies have been demonstrated the significance of gut microbiota in the CRC pathogenesis.^3^ Various bacteria such as *Fusobacterium nucleatum*, *Enterotoxigenic Bacteroides fragilis*, *PKS*+ *Escherichia coli* and *Campylobacter jejuni* have been also confirmed to be associated with CRC in recent years.^4^, ^5^, ^6^, ^7^

In addition to the role of gut microbiota, increasing studies have focused on the oral microbiota in the development of CRC.^8^ Oral‐typical microbes may influence CRC through various mechanism, including the colonisation of oral bacteria in the gut, promoting tumour cell proliferation and invasion, and remodelling the tumour microenvironment. Previous study has demonstrated that periodontal pathogen *Porphyromonas gingivalis* promoted colorectal tumourigenesis by recruiting myeloid cells and creating a proinflammatory tumour microenvironment.^9^ Another oral microbiota member *Peptostreptococcus anaerobius* has been found to induce dysplasia of colon cell and mediated resistance to anti‐PD1 therapy in CRC.^8^, ^10^ Furthermore, a most recent study revealed an enrichment of oral‐typical microbes in CRC through an integrated analysis of 12 metagenomic datasets.^11^ In particular, *Parvimonas micra* was already increased in stage I and late‐stage CRC was also found to be enriched in oral‐derived species, such as *P. micra* and *Hungatella hathewayi*.^11^ Although the overabundance of these bacteria in the gut niche may be an aetiological factor in CRC, their role in carcinogenesis remains to be established.

Gut dysbiosis has been demonstrated to be associated with an alteration of metabolic pool. Limit evidence was found to evaluate the impact of oral‐typical microbes and their metabolites during CRC tumourigenesis. *P. gingivalis* identified in CRC patients promote tumourigenesis via butyrate secretion.^12^ Trans‐3‐indoleacrylic acid derived from *P. anaerobius* has been found to act as an endogenous ligand of an aryl hydrocarbon receptor (AHR) to facilitate colorectal carcinogenesis.^13^ However, an integrative analysis of oral‐typical microbes and their underlying mechanism is far from clear.

In this study, a CRC cohort for an integrative analysis of metagenomics and metabolomics was established. We identified an accumulation of *P. micra* in faeces from CRC patients. The role of *P. micra* and its metabolite in CRC carcinogenesis has been also confirmed in vivo and in vitro. These findings provide diagnostic and therapeutic targets for treating CRC.

## 2 METHODS

### 2.1 Human subjects

Faecal samples were obtained from patients diagnosed with CRC. Volunteers who showed no evidence of CRC based on nonspecific symptoms, endoscopic evaluation and histopathological examination were classified as healthy controls. Clinical parameters were collected (Table S1). Exclusion criteria included: unwillingness or inability to provide tissue samples, antibiotic use within the 2 weeks prior to enrollment, acute gastrointestinal infection or perforation, pregnancy, known bleeding disorders or a diagnosis of ileus. Written informed consent was obtained from all participants during their preoperative visit. The study protocol was approved by the Human Medical Ethics Committee of the Sixth Affiliated Hospital of Sun Yat‐Sen University (approval no. 2023ZSLYEC‐114).

### 2.2 Mice

Six‐ to eight‐week‐old male mice were used in this study. C57BL/6J wild‐type (WT), *Apc* ^Min/+^ and *Ahr^−/−^* mice were obtained from GemPharmatech Co., Ltd. All animals were maintained under specific pathogen‐free conditions at the Sixth Affiliated Hospital of Sun Yat‐Sen University and Guangzhou Ruige Biological Technology Co., Ltd., with a 12‐h light/dark cycle and provided free access to water and standard rodent diet. All experimental procedures were conducted in compliance with protocols approved by the Institutional Animal Care and Use Committee of Sun Yat‐Sen University and Guangzhou Ruige Biological Technology Co., Ltd.

### 2.3 Cell culture

The cell lines were maintained in Gibco Dulbecco's Modified Eagle Medium or RPMI‐1640 medium supplemented with 10% foetal bovine serum (GIBCO) and 1% penicillin‒streptomycin (GIBCO), and incubated at 37°C under a humidified atmosphere of 5% CO_2_.

### 2.4 Bacteria culture

Cultivation media were pre‐reduced for 48 h in an anaerobic chamber (90% N_2_, 5% CO_2_, 5% H_2_) prior to inoculation. *P. micra* was isolated from CRC faecal samples with Columbia Blood Agar Plate. Bacterial cultures were grown 3 days at 37°C under anaerobic conditions using a chamber (model CAW1100, Guangzhou Huanghe Instrument Technology Co., Ltd.). Bacterial isolation and identification were performed using a colony picker (RapidPick SP, Guangzhou Jinke Chian Technology Co., Ltd.), a detection system (CMI‐1600, Guangzhou Chengyi Imp & Exp Co., Ltd.) and a spiral dilution system (Easy spiral dilute, Zhongke Scientific & Technical Co., Ltd.).

### 2.5 Transgenic *Apc^Min/+^* mice model

To confirm the role of *P. micra* in CRC, we established an intestinal adenoma spontaneous mice model with *Apc^Min/+^* mice. *Apc^Min/+^* mice were exposed to 2% dextran sulphate sodium salt (DSS) for 1 week after 2‐week treatment of *P. micra* (1 × 10^9^ CFU/200 µL) or phosphate‐buffered saline (PBS). Subsequently, *Apc^Min/+^* mice were gavaged with *P. micra* (1 × 10^9^ CFU/200 µL) or PBS three times a week for 7 weeks. To evaluate the role of phenyllactic acid (PLA) in CRC, *Apc^Min/+^* mice were gavage with PLA (10mg/kg) or PBS for 2 weeks and subsequently exposed to 2% DSS for 1 week. In following 7 weeks, *Apc^Min/+^* mice were gavaged with PLA or PBS three times a week.

### 2.6 Azoxymethane/DSS mice model

To establish the azoxymethane (AOM)/DSS mice model, C57BL/6J WT mice and *Ahr^−/−^* mice were intraperitoneally injected with AOM (10 mg/kg) followed by DSS administration. In AOM/DSS treatment, mice were injected by AOM at the first day and subsequently challenged with 2% DSS for 1 week followed by normal drinking for 1 week. Mice were sacrificed after three cycles of DSS intervention. Mice were gavaged with PLA (10 mg/kg) or *P. micra* (1 × 10^9^ CFU/200 µL) three times a week throughout the experiment.

### 2.7 Patient‐derived colorectal tumour organoids

CRC tumour tissues were collected and washed five times with cold PBS (5 min per wash) as previously described.^14^ The tissues were minced into a fine paste using sterile scissors in a petri dish and then digested with a collagenase solution (RPMI‐1640 medium containing 5% FBS, .5 mg/mL Collagenase IV and 1 mg/mL DNase I) at 37°C for 30‒60 min. After digestion, the suspension was centrifuged at 300‒500 × *g* for 5 min to pellet the tumour cells. The cell pellet was resuspended and seeded into Matrigel (ABW) in pre‐warmed 24‐well flat‐bottom plates. Following a 20‐min incubation at 37°C in a 5% CO_2_ incubator, 500 µL of human reduced‐serum culture medium was gently overlaid. The medium was routinely replaced every 2‒3 days. Tumour organoids typically became visible after 48 h of culture.

### 2.8 Quantitative real‐time PCR

Cells and tissues were collected, and total RNA was extracted using the Total RNA Kit (R323‐01, Vazyme). cDNA was synthesised with the Hiscript III RT SuperMix for qPCR (gDNA wiper) (R323‐01, Vazyme). Quantitative real‐time PCR was performed on an Applied Biosystems 7500 Real‐Time PCR System using SYBR Green Real‐Time PCR Master Mix (QPK‐201, Toyobo). The primer sequences used in this study are provided in Table S2.

### 2.9 Immunofluorescence analysis

Tissue sections were freshly prepared, fixed in 10% formalin, and embedded in paraffin. Staining was performed on 4 µm paraffin sections. After deparaffinisation with xylene and rehydration through an ethanol series, antigen retrieval was conducted by incubating slides in boiling sodium citrate buffer (10 mM, pH 6.0) for 10 min. Sections were then blocked with PBS containing .6% Triton X‐100 for 15 min, followed by three 5‐min washes in .1% Tween 20 in PBS (PBST). Primary antibody incubation was carried out overnight at 4°C. After washing three times with PBST as above, slides were incubated with secondary antibodies for 30 min at room temperature. Finally, tissues were mounted using ProLong Antifade Mountant with DAPI, coverslipped, and sealed to prevent drying. Imaging was performed on a Leica laser scanning confocal microscope, and quantification was conducted using ImageJ.

### 2.10 Heterologous expression of *pdhD* in *E. coli*

The *pdhD* gene from *P. micra* (Table S2) was codon‐optimised for *E. coli* expression using the online tool OPTIMIZER (http://genomes.urv.es/OPTIMIZER/) combined with OptimumGene (Genscript) and manual refinement to adjust rare codons, GC content, and remove negative *cis*‐elements and repeats. The optimised gene was cloned into the pET15b vector for overexpression. Chemically competent *E. coli* *BL21*(DE3) cells were transformed with the resulting plasmid via heat shock to generate the *E. coli* *BL21‐pdhD* strain. For control, the empty pET15b vector was similarly transformed to produce *E. coli* *BL21* WT. Transformed colonies were selected on plates containing 100 µg/mL ampicillin at 37°C for 10 h. A single colony was inoculated into LB medium and grown overnight. The overnight culture was used to inoculate 50 mL of fresh LB medium, which was incubated at 37°C with shaking at 220 rpm. When the OD600 reached .6‒.8, *pdhD* expression was induced by adding 1 mM Isopropyl β‐D‐Thiogalactopyranoside (IPTG).

### 2.11 Metagenomic analysis

Metagenomic analysis of faecal samples from CRC patients and healthy controls was performed by Majorbio Biotechnology Co., Ltd. For metagenomic analysis, raw FASTQ files were quality‐controlled using KneadData (v0.7.7), which integrates Trimmomatic and Bowtie2 for adapter trimming and host DNA removal.^15^, ^16^ Trimmomatic was run with default parameters (‘SLIDINGWINDOW:4:20 MINLEN:70’), where the minimum length threshold was set to 70% of the original read length. The human reference genome GRCh38 was used for de‐hosting. Taxonomic classification and abundance estimation were performed with Kraken2 and Bracken, respectively.^17^, ^18^ Functional profiling was conducted using HUMAnN2 (UniRef90 database), which computes pathway‐stratified and organism‐specific gene family abundances based on the ChocoPhlAn and UniRef90 databases. Associations between clinical parameters and microbial features were evaluated using linear mixed‐effects models implemented in the R package MaAsLin3. The resulting *p*‐values and beta coefficients reflect the significance and effect size of dose‒response relationships between clinical variables and microbial abundance.

### 2.12 Untargeted metabolomics

Untargeted metabolomic analysis of faecal samples from CRC patients and healthy controls was performed by Maiwei Metabolic Biotechnology Co., Ltd.^19^, ^20^ Samples stored at −80°C were thawed on ice and vortexed for 10 s. A 50 µL aliquot of each sample was mixed with 300 µL of extraction solution (acetonitrile:methanol = 1:4, v/v) containing internal standards in a 2 mL microcentrifuge tube. After vortexing for 3 min, the mixture was centrifuged at 12 000 rpm for 10 min at 4°C. The supernatant (200 µL) was transferred, incubated at −20°C for 30 min, and centrifuged again at 12 000 rpm for 3 min at 4°C. Finally, 180 µL of the supernatant was collected for LC‒MS analysis. LC‐MS/MS was performed on a QTRAP system (SCIEX) equipped with an electrospray ionisation (ESI) source operating in both positive and negative modes. Data acquisition was controlled by Analyst 1.6.3 software. The ESI parameters were set as follows: source temperature 500°C; ion spray voltage 5500 V (positive) and −4500 V (negative); ion source gas I (GSI), gas II (GSII) and curtain gas (CUR) at 55, 60 and 25.0 psi, respectively; collision gas (CAD) set to high. System tuning and calibration were conducted using 10 and 100 µmol/L polypropylene glycol solutions in QQQ and LIT modes, respectively. Metabolites were monitored via multiple reaction monitoring transitions according to their retention times. Quantification was carried out using MetWare (http://www.metware.cn/) based on the MWDB metabolome database.

### 2.13 Quantification and statistical analysis

All statistical analyses were performed using R (version 4.3) or GraphPad Prism 10 (GraphPad Software). In vitro experiment was repeated three times. Differences between two groups were assessed by Student's *t*‐test (parametric) or the Mann‒Whitney *U*‐test (non‐parametric). For comparisons among three or more groups, one‐way analysis of variance (parametric) or the Kruskal‒Wallis test (non‐parametric) was applied. A threshold of *p* < .05 (adjusted where applicable) was considered statistically significant. Significance levels are denoted with asterisks as follows: ^*^ *p* < .05; ^**^ *p* < .01; ^***^ *p* < .001; ^****^ *p* < .0001; ‘ns’ indicates non‐significant results.

## 3 RESULTS

### 3.1 Higher abundance of *P. micra* in faeces is associated with CRC development

**FIGURE 1:**
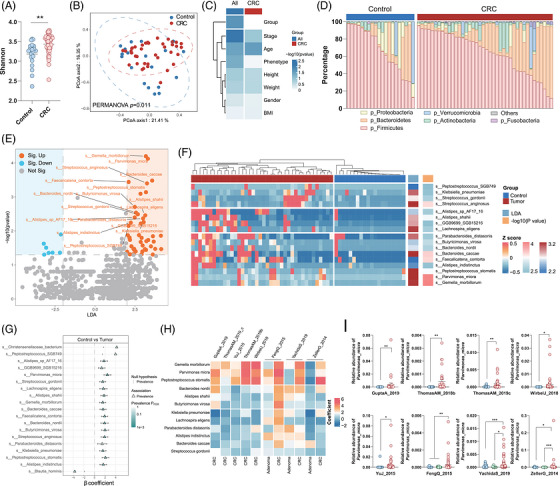
Higher abundance of *Parvimonas micra* in faeces from colorectal cancer (CRC) patients. (A) Microbial diversity of faecal microbiome from CRC patients and healthy controls. (B) Principal component analysis of microbial structure in faecal samples from CRC patients and healthy controls. (C) Variance of different clinical parameters for metagenomics. (D) Proportion of different phylum in faecal samples from CRC patients and healthy controls. (E) Differential microbiota in faecal samples between CRC patients and healthy controls shown by volcano plot. (F) Heatmap showing the most enrichment of faecal microbiota in faeces from CRC patients. (G) Association of specific faecal microbiota and CRC calculated by MaAsLin3. (H) Association of faecal microbiota and CRC in eight public metagenomic datasets. (I) Relative abundance of *P. micra* in faecal samples from eight public metagenomic datasets. Mean ± SEM, ^*^ *p* < .05; ^**^ *p* < .01; ^***^ *p* < .001 compared using unpaired Student's *t*‐test (two‐tailed).

To characterise the relationship between faecal microbiome and CRC, we established a clinical cohort where 40 CRC patients and 20 healthy controls were recruited, and faecal samples were collected for metagenomics analysis (Table S1). Significant higher *α* diversity was found in CRC patents (Figure 1A). Principal component analysis showed that microbial structure from CRC patients was significantly separated from healthy controls (Figure 1B). In addition to the group of disease, several clinical parameters such as age and tumour stage were associated with the alteration of microbiome (Figure 1C). The trends of general microbial structure in CRC patients were characterised with an increased proportion of Bacteroidetes and Actinobacteria, while the proportions of Firmicutes and Proteobacteria were decreased (Figure 1D). Then, we applied linear discriminant analysis of effect size (LEfSe) to detect marked difference in the predominance of bacterial communities between CRC and healthy controls. There were 49 species that significantly accumulated in faeces from CRC patients, while seven species were downregulated (Figure 1E). Specifically, 17 species such as *Gemella morbillorum*, *P. micra*, *Bacteroides caccae*, *Streptococcus anginosus*, *Parabacteroides distasonis*, *Peptostreptococcus stomatis* and *Streptococcus gordonii* were significantly enriched in faeces from CRC patients (Figure 1F). And *G. morbillorum* and *P. micra* were the ones that displayed the most significant changes in CRC (Figure 1F). To further clarify the role of specific microbiota in CRC, we applied multivariable linear models with MaAsLin3 to evaluate the relationship between microbiota and CRC. The presence of *P. micra* exhibited the most significant relationship with the occurrence of CRC (Figure 1G).

To validate the role of *P. micra* in CRC development, we analysed 1379 faecal metagenomes from eight public cohorts.^21^, ^22^, ^23^, ^24^, ^25^, ^26^, ^27^ Multivariable linear model analysis of these eight cohorts revealed that the presence of *P. micra* was significantly associated with the development of CRC (Figure 1H). Moreover, significantly higher abundance of *P. micra* was found in CRC patients from five public cohorts (Figure 1I). Compared with those patients with adenoma, an obvious increase in *P. micra* was also observed in CRC patients from three public cohorts (Figure 1I). Overall, these results suggest that increased colonisation of *P. micra* was associated with the development of CRC.

### 3.2 Upregulation of PLA is involved in CRC

**FIGURE 2:**
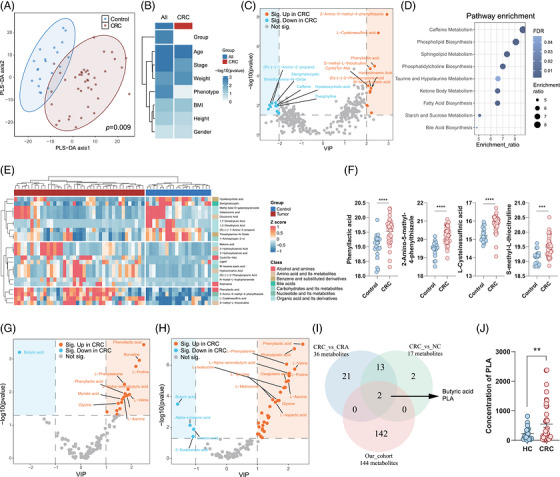
Accumulation of phenyllactic acid (PLA) in faeces from colorectal cancer (CRC) patients. (A) Distributions of faecal metabolites from CRC patients and healthy controls. (B) Variance of different clinical parameters for metabolomics. (C) Differential metabolites in faecal samples between CRC patients and healthy controls shown by volcano plot. (D) Pathway enrichment analysis of the differential metabolites. (E) Heatmap showing the differential metabolites which exhibited the most significant changes. (F) Concentration of different significantly altered metabolites PLA, 2‐amino‐5‐methyl‐4‐phenylthiazole, L‐cysteinesulphinic acid and S‐methyl‐L‐thiocitrulline). (G) Differential metabolites in faecal samples between CRC patients and normal controls from public dataset. (H) Differential metabolites in faecal samples between CRC patients and patients with colorectal adenoma from public dataset. (I) Venn diagram showing the metabolites that were significantly upregulated in all three datasets. (J) Concentration of PLA in faecal samples from CRC and healthy control. (F and J) Mean ± SEM, ^*^ *p* < .05; ^**^ *p* < .01; ^***^ *p* < .001 compared using unpaired Student's *t*‐test (two‐tailed).

To explore how microbiota mediate the CRC development, faecal samples in our clinical cohort were subsequently collected for untargeted metabolomics. Consistent with the results observed in taxonomic profile, alteration of overall metabolite pool from CRC patients exhibited an obviously separation from healthy controls (Figure 2A). Several clinical parameters such as disease, age, tumour stage and weight were associated with the alteration of metabolome (Figure 2B). There were 14 metabolites that significantly upregulated in faeces from CRC patients, while 16 metabolites were downregulated (Figure 2C). Pathway analysis showed that the differential metabolites were associated with several pathways such as phospholipid biosynthesis, sphingolipid metabolism, phosphatidylcholine biosynthesis, fatty acid biosynthesis, ketone body metabolism, bile acid biosynthesis, starch and sucrose metabolism (Figure 2D). Specifically, 24 metabolites with the most significant changes (VIP > 2, *p *< .05) were identified (Figure 2E). Several metabolites associated with the organic acid such as methyl beta‐D‐galactopyranoside, galacturonic acid, 1,7‐dimethyluric acid, 1,3‐dimethyluric acid and (R)‐(‒)‐1‐amino‐2‐propanol were obviously downregulated in faeces from CRC patients (Figure 2E). Four metabolites, including PLA, 2‐amino‐5‐methyl‐4‐phenylthiazole, L‐cysteinesulphinic acid and S‐methyl‐L‐thiocitrulline, exhibited the most significant increase in CRC (Figure 2F). To further identify the key metabolite in CRC development, we integrated the public metabolic datasets.^28^ Compared with healthy control or adenoma, most of the differential metabolites were upregulated in CRC patients (Figure 2G,H). Although distinct patterns of metabolic pool were observed in different cohorts, only two metabolites, butyric acid and PLA, were the common metabolites that were significantly increased in three cohorts (Figure 2I). More importantly, the increasing changes of PLA was the most pronounced in three cohorts. To validate the concentration of PLA in CRC, we established another clinical cohort and faecal samples were collected for LC‒MS. Significantly higher concentration of PLA was confirmed in faecal samples from CRC patients (Figure 2J). Collectively, these data identified that PLA may be the key metabolite to mediate the CRC development.

### 3.3 *P. micra* promotes the CRC development via biosynthesis of PLA

**FIGURE 3:**
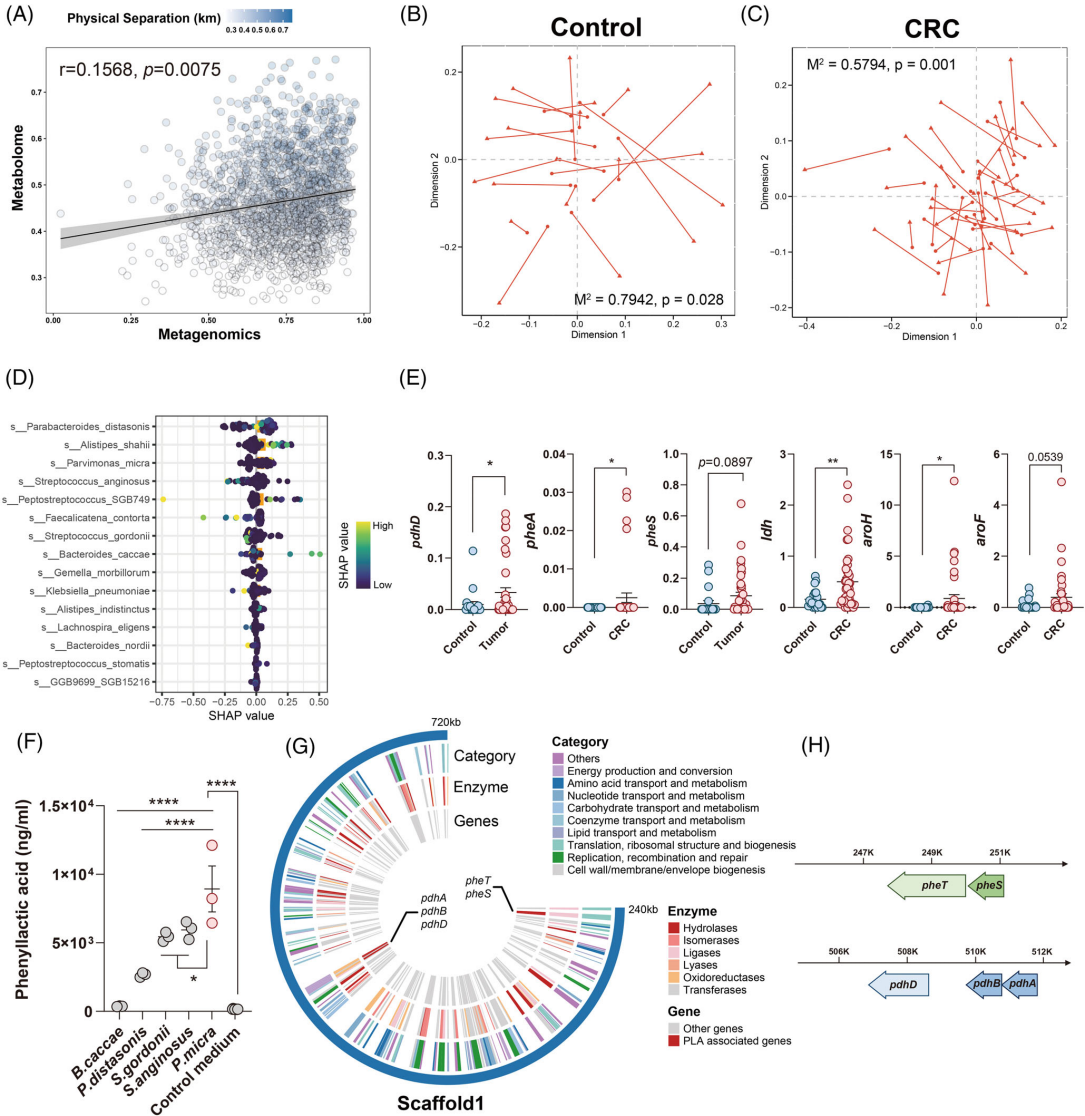
*Parvimonas micra* was able to mediate the biosynthesis of phenyllactic acid (PLA). (A) Mantel tests quantifying variance explained between faecal metagenome and faecal metabolome. (B and C) Procrustes analysis connecting the faecal microbiomes and faecal metabolome from healthy controls (B) and patients with CRC (C). (D) The importance of each differential species for PLA biosynthesis ranking by the average absolute SHAP value. (E) Relative abundance of microbial genes associated with PLA biosynthesis. (F) Concentration of PLA in the supernatant from different isolated species. (G and H) Schematic representation of the PLA‐associated genes in the genome of *P. micra*. (E) Mean ± SEM, ^*^ *p* < .05; ^**^ *p* < .01; ^***^ *p* < .001 compared using unpaired Student's *t*‐test (two‐tailed). (F) Mean ± SEM, ^*^ *p* < .05; ^**^ *p* < .01; ^***^ *p* < .001; ^****^ *p* < .0001 compared using one‐way analysis of variance (ANOVA) comparisons test.

To reveal the interaction between faecal metabolites and faecal microbiome, we applied mantel test to quantify the variance explained between faecal microbiome and metabolome. Faecal metagenomics was tightly coupled with faecal metabolome (*r* = .1568, *p *= .0075) (Figure 3A). Moreover, procrustes analysis based on Bray‒Curtis distances revealed that a better fit and significant correlation between the microbial community composition and metabolic pool was found in CRC patients (CRC: *M* ^2^ = .5794, *p* = .001; healthy control: *M* ^2^ = .7942, *p* = .028) (Figure 3B,C). These results suggest a close relationship between the gut metabolites and microbiome during CRC development.

But how microbiota modulate the synthesis of PLA? To better understand which microbiota contributed to the alteration of PLA, we established the gradient‐boosted decision tree model and the importance of different species in PLA biosynthesis were calculated with SHAP value. According to the importance ranking of the average absolute SHAP value, *P. micra* was assessed as one of the important variables (Figure 3D). We subsequently evaluate the microbial genes that were associated with PLA biosynthesis. Several genes such as *pdhD*, *pheA*, *pheS*, *ldh*, *aroH* and *aroF* were increased in faeces from CRC patients (Figure 3E). To validate these relationships, we anaerobically isolated five microbial species (*P. micra*, *B. caccae*, *P. distasonis*, *S. anginosus* and *S. gordonii*) in faeces from CRC patients, and detected the concentration of PLA in their supernatant. As expected, significantly higher level of PLA was observed in the supernatant from *P. micra* (Figure 3F). To further characterise the functions of *P. micra* in PLA biosynthesis, whole‐genome sequencing of the isolated *P. micra* was conducted to detect the expression of PLA‐associated genes (Figure 3G). Specifically, a series of PLA‐associated genes such as *pdhD* were detected in *P. micra* (Figure 3H), suggesting the potential involvement of *P. micra* in PLA biosynthesis. These data provided a hypothesis that *P. micra* could modulate the biosynthesis of PLA via *pdhD* gene, leading to the CRC development.

### 3.4 Role of *P. micra* and its metabolites in the CRC mice model

**FIGURE 4:**
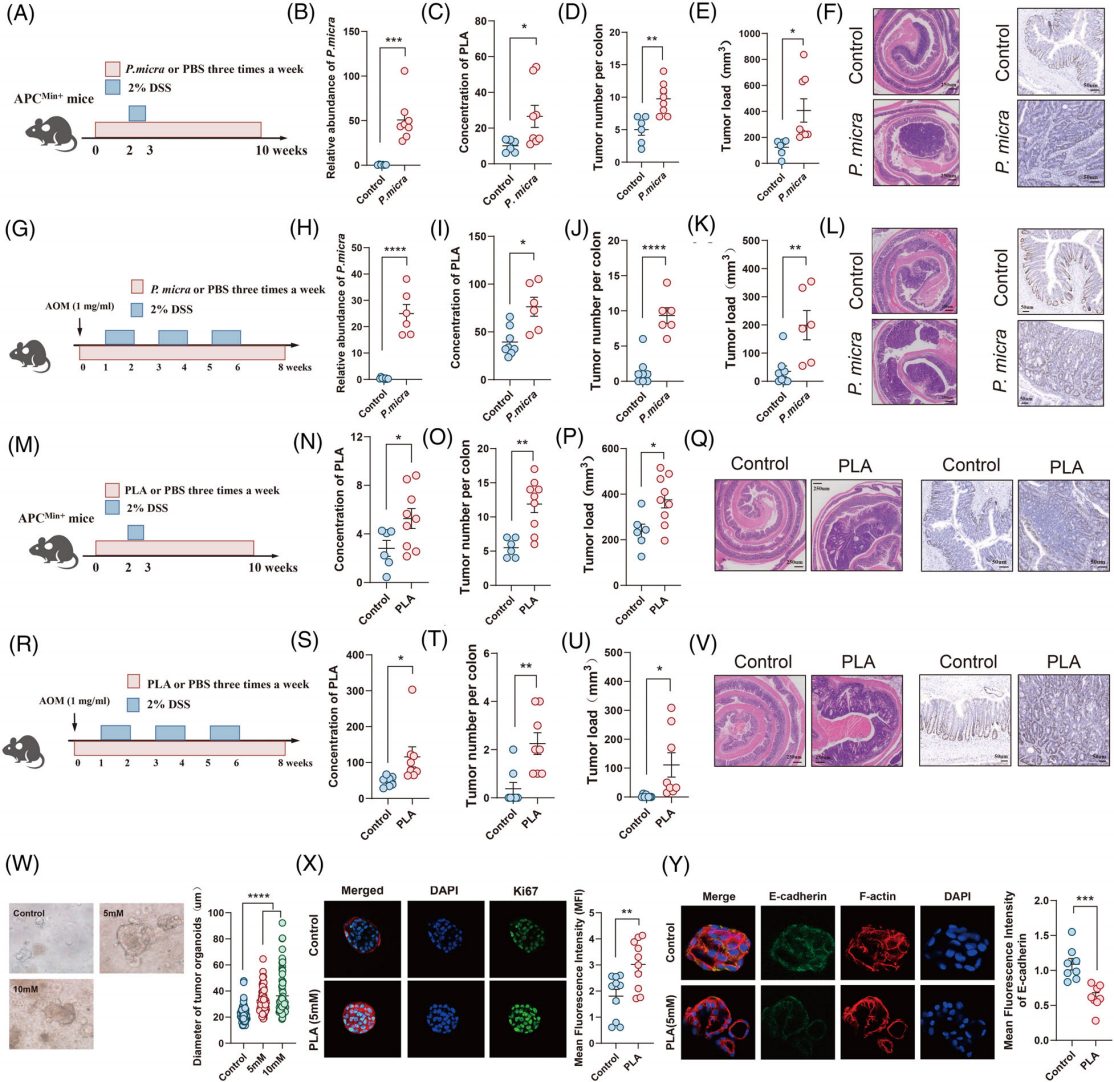
Both of *Parvimonas micra* and phenyllactic acid (PLA) promote colorectal cancer (CRC) carcinogenesis in mice model. (A) Schematic diagram showing the process of *Apc* ^Min/+^ mice model treated with *P. micra* and phosphate‐buffered saline (PBS) control. (B) Relative abundance of *P. micra* in faeces from mice treated with *P. micra* (*n* = 8) and PBS control (*n* = 6). (C) Concentration of PLA in faeces from mice treated with *P. micra* and PBS control. (D and E) Tumour number per colon and tumour load from mice treated with *P. micra* and PBS control. (F) Representative images of colonic histopathological images and Ki67 staining. (G) Schematic diagram showing the process of azoxymethane (AOM)/dextran sulphate sodium salt (DSS) mice model treated with *P. micra* (*n* = 6) and PBS control (*n* = 8). (H) Relative abundance of *P. micra* in faeces from mice treated with *P. micra* and PBS control. (I) Concentration of PLA in faeces from mice treated with *P. micra* and PBS control. (J and K) Tumour number per colon and tumour load from mice treated with *P. micra* and PBS control. (L) Representative images of colonic histopathological images and Ki67 staining. (M) Schematic diagram showing the process of *Apc* ^Min/+^ mice model treated with PLA (*n* = 9) and PBS control (*n* = 6). (N) Concentration of PLA in faeces from mice treated with PLA and PBS control. (O and P) Tumour number per colon and tumour load from mice treated with PLA and PBS control. (Q) Representative images of colonic histopathological images and Ki67 staining. (R) Schematic diagram showing the process of AOM/DSS mice model treated with PLA (*n* = 8) and PBS control (*n* = 7). (S) Concentration of PLA in faeces from mice treated with PLA and PBS control. (T and U) Tumour number per colon and tumour load from mice treated with PLA and PBS control. (V) Representative images of colonic histopathological images and Ki67 staining. (W) Representative images of CRC organoid treated with PBS and PLA (5 and 10 mM), and diameter of tumour organoids. (X and Y) Representative images of CRC organoid stained by Ki‐67 and E‐cadherin, and mean fluorescence intensity of CRC organoid treated with PBS and PLA. (B‒E, H‒K, N‒P, S‒U, X and Y) Mean ± SEM, ^*^ *p* < .05; ^**^ *p* < .01; ^***^ *p* < .001 compared using unpaired Student's *t*‐test (two‐tailed). (W) Mean ± SEM, ^*^ *p* < .05; ^**^ *p* < .01; ^***^ *p* < .001; ^****^ *p* < .0001 compared using one‐way analysis of variance (ANOVA) comparisons test.

To validate the role of *P. micra* in CRC, we established a CRC mice model with *Apc* ^Min/+^ mice. After gavage with *P. micra* for 2 weeks, *Apc* ^Min/+^ mice were exposed to 2% DSS for 1 week and followed by 7‐week treatment with *P. micra* (Figure 4A). Higher colonisation of *P. micra* was detected in mice treated with *P. micra* (Figure 4B). Colonisation of *P. micra* increased the concentration of PLA in faecal samples (Figure 4C). Macroscopic evaluation of the colon revealed a significantly higher tumour load and proliferation in mice treated with *P. micra* (Figure 4D‒F). We further established a CRC tumourigenesis mice model using carcinogen AOM and 2% DSS (Figure 4G). Mice were gavage with *P. micra* three times a week and higher colonisation of *P. micra* was observed (Figure 4H). Higher colonisation of *P. micra* was associated with higher concentration of PLA in faecal samples (Figure 4I). Similarly, *P. micra* was able to induce carcinogenesis in AOM/DSS mice model (Figure 4J‒L).

To further clarify the impact of PLA on CRC mice model, *Apc* ^Min/+^ mice were gavaged with PLA as shown in Figure 4M. We found a significantly higher concentration of PLA, increased colon tumour size and tumour load in mice gavaged with PLA (Figure 4N‒P). Histological assessment also confirmed more colon tumours and the increased proportion of proliferating cells in mice treated with PLA (Figure 4Q). Moreover, we also established a AOM/DSS mice model and mice were gavage with PLA three times a week (Figure 4R). As expected, mice treated PLA exhibited a significantly higher colon tumour size and tumour load (Figure 4S‒V). In addition to different mice models, we subsequently constructed a CRC organoid model. Significantly higher diameter of CRC organoids was observed after treatment of PLA (Figure 4W). Higher cellular proliferation stained by Ki‐67 were observed in CRC organoid treated with PLA (Figure 4X). Lower expression of E‐cadherin was also detected in PLA‐treated CRC organoid, indicating a higher invasiveness (Figure 4Y). Collectively, these data revealed that *P. micra* was able to mediate the development of CRC via upregulation of PLA.

### 3.5 PLA from *P. micra* induces DNA damage to mediate CRC development

**FIGURE 5:**
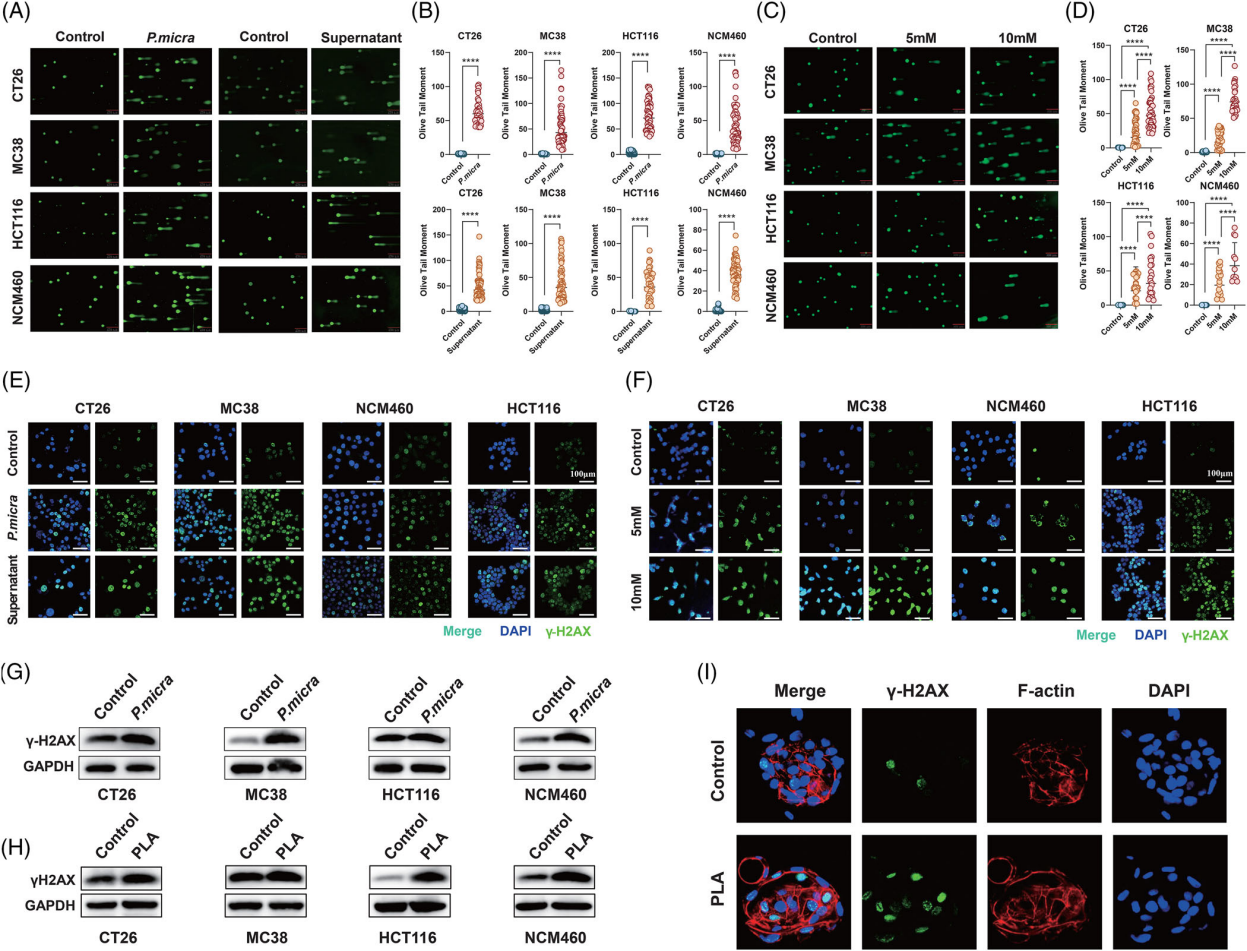
Phenyllactic acid (PLA) from *Parvimonas micra*‐induced DNA damage to mediate colorectal cancer (CRC) development. (A and B) Comet assay of the CT26, MC38, HCT116 and NCM460 cell line treated with *P. micra*, supernatant of *P. micra* or phosphate‐buffered saline (PBS) control. (C and D) Comet assay of the CT26, MC38, HCT116 and NCM460 cell line treated with PLA or PBS control. (E) Immunofluorescence analysis of γ‐H2AX in CT26, MC38, HCT116 and NCM460 cell line treated with *P. micra*, supernatant of *P. micra* or PBS control. (F) Immunofluorescence analysis of γ‐H2AX in CT26, MC38, HCT116 and NCM460 cell line treated with PLA or PBS control. (G) Protein expression of γ‐H2AX in CT26, MC38, HCT116 and NCM460 cell line treated with *P. micra* or PBS control. (H) Protein expression of γ‐H2AX in CT26, MC38, HCT116 and NCM460 cell line treated with PLA or PBS control. (I) Representative images of CRC organoid stained by γ‐H2AX and F‐actin. (B) Mean ± SEM, ^*^ *p* < .05; ^**^ *p* < .01; ^***^ *p* < .001 compared using unpaired Student's *t*‐test (two‐tailed). (D) Mean ± SEM, ^*^ *p* < .05; ^**^ *p* < .01; ^***^ *p* < .001; ^****^ *p* < .0001 compared using one‐way analysis of variance (ANOVA) comparisons test.

Microbial‐induced DNA damage in epithelial cells was one of the important mechanisms in CRC development.^29^ Thus, murine colon carcinoma cell line CT26 and MC38, human colon cancer cell line HCT116 and normal human colon mucosal epithelial cell line NCM460 were exposed to *P. micra* and PLA. We found that *P. micra* and PLA significantly reduced the apoptosis and PLA could increase the proliferation of these four different cell lines (Figure S1A‒C). DNA damage in different cell lines was subsequently evaluated. DNA damage detected by comet assay showed that both *P. micra* and its supernatant could induce DNA damage in different cell lines (Figure 5A,B). Similar trends that higher olive tail moment were observed in different cell lines treated with PLA (Figure 5C,D). Moreover, immunofluorescence also revealed that *P. micra* and PLA increased γ‐H2AX, a surrogate marker for DNA damage in all different cell lines, when compared with untreated cells (Figures 5E,F and S1D,E). Quantification of transcript and protein level further validated the significant upregulation of γ‐H2AX in different cell lines after treated with *P. micra*, supernatant of *P. micra* and PLA (Figures 5G,H and S1F‒I). To further validate the DNA damage induced by *P. micra* and PLA, we evaluated the expression of γ‐H2AX in CRC organoid and the intestinal tissues from different mice models. Higher expression of γ‐H2AX was detected in CRC organoid treated with PLA (Figure 5I). Consistent with the CRC organoid, higher expression of γ‐H2AX was also observed in intestinal tissues from mice treated with *P. micra* and PLA in different mice models (Figure S2A‒D). Collectively, these data demonstrated that the roles of *P. micra* and PLA in CRC development were associated with the induction of DNA damage.

### 3.6 *P*. *micra* modulates the biosynthesis of PLA via *pdhD* gene

**FIGURE 6:**
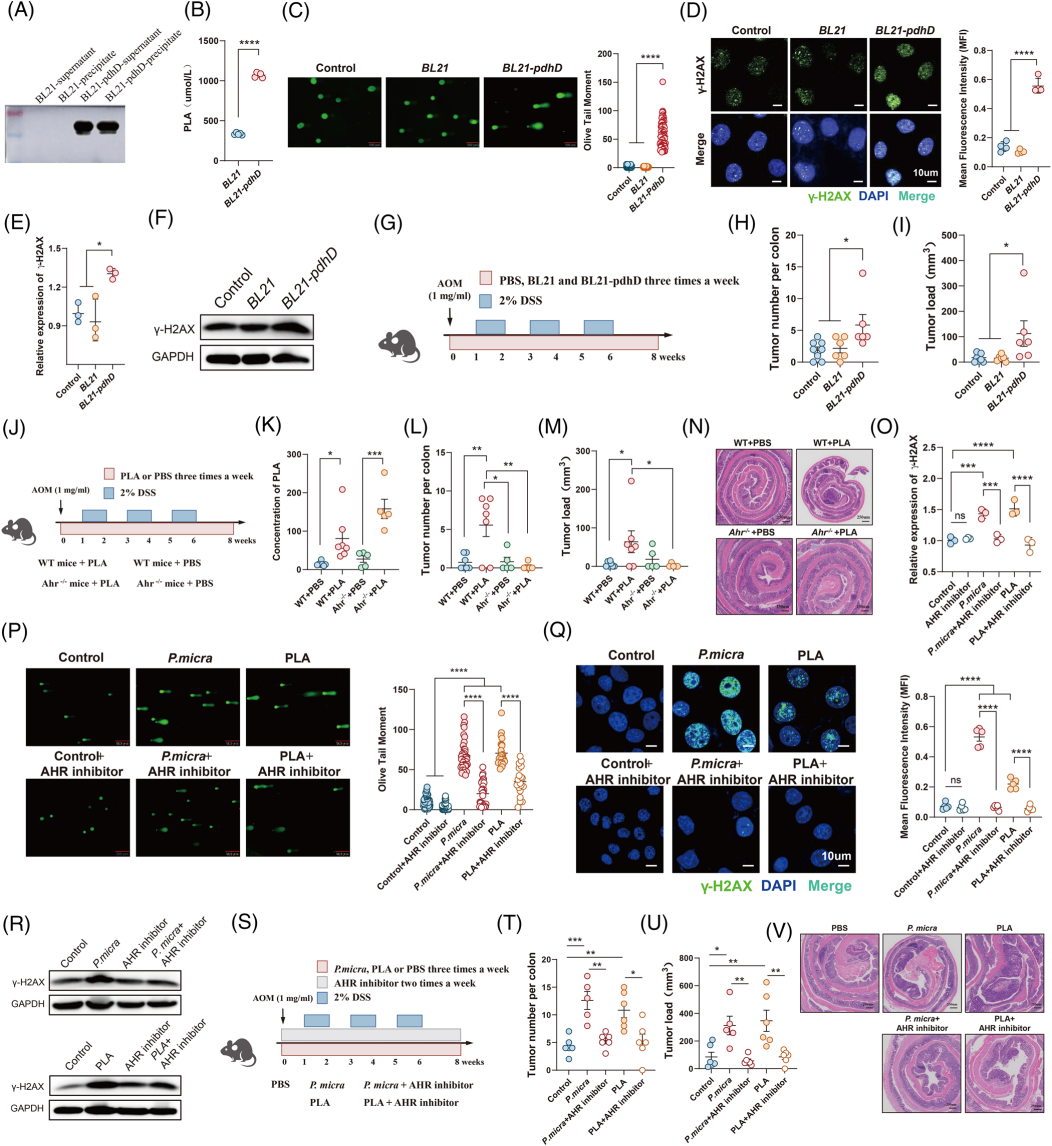
*Parvimonas micra*‐derived phenyllactic acid (PLA) mediate colorectal cancer (CRC) carcinogenesis via aryl hydrocarbon receptor (AHR) signalling. (A) Protein expression of *pdhD* in *Escherichia coli* strain *BL21* by Western blotting. (B) Concentration of PLA in the supernatant of *BL21* and *BL21‐pdhD*. (C) Comet assay of the NCM460 cell line treated with supernatant from *BL21*, *BL21‐pdhD* or phosphate‐buffered saline (PBS) control. (D) Immunofluorescence analysis of γ‐H2AX in NCM460 cell line treated with supernatant from *BL21*, *BL21‐pdhD* or PBS control. (E and F) Transcript and protein level of γ‐H2AX in NCM460 cell line treated with supernatant from *BL21*, *BL21‐pdhD* or PBS control. (G) Schematic diagram showing the process of azoxymethane (AOM)/dextran sulphate sodium salt (DSS) mice model treated with *BL21* (*n* = 6), *BL21‐pdhD* (*n* = 6) or PBS control (*n* = 8). (H and I) Tumour number per colon and tumour load from mice treated with *BL21*, *BL21‐pdhD* or PBS control. (J) Schematic diagram showing the process of AOM/DSS mice model: WT + PBS (*n* = 7), WT + PLA (*n* = 7), *Ahr^−/−^* + PBS (*n* = 5) and *Ahr^−/−^* + PLA (*n* = 5). (K) Concentration of PLA in faeces from *Ahr^−/−^* mice and WT mice treated with PLA and PBS control. (L and M) Tumour number per colon and tumour load from mice treated with PLA and PBS control. (N) Representative images of colonic histopathological images. (O) Transcript level of γ‐H2AX in NCM460 cell line treated with AHR inhibitor. (P) Comet assay of the NCM460 cell line treated with *P. micra*, PLA, *P. micra *+ AHR inhibitor, PLA + AHR inhibitor or PBS control. (Q) Immunofluorescence analysis of γ‐H2AX in NCM460 cell line treated with *P. micra*, PLA, *P. micra *+ AHR inhibitor, PLA + AHR inhibitor or PBS control. (R) Protein level of γ‐H2AX in NCM460 cell line treated with AHR inhibitor. (S) Schematic diagram showing the process of AOM/DSS mice model treated with *P. micra* (*n* = 5), PLA (*n* = 6), *P. micra *+ AHR inhibitor (*n* = 6), PLA + AHR inhibitor (*n* = 6) or PBS control (*n* = 6). (T and U) Tumour number per colon and tumour load from mice treated with *P. micra*, PLA, *P. micra* + AHR inhibitor, PLA + AHR inhibitor or PBS control. (V) Representative images of colonic histopathological images. (B) Mean ± SEM, ^*^ *p* < .05; ^**^ *p* < .01; ^***^ *p* < .001 compared using unpaired Student's *t*‐test (two‐tailed). (C‒E, H‒I, K‒M, O‒P and T‒U) Mean ± SEM, ^*^ *p* < .05; ^**^ *p* < .01; ^***^ *p* < .001; ^****^ *p* < .0001 compared using one‐way analysis of variance (ANOVA) comparisons test.

Consistent with the colonisation of *P. micra*, significantly higher level of *pdhD* gene were detected in mice treated with *P. micra* in different CRC mice model (Figure S3A). *PdhD* is a gene encoding dihydrolipoyl dehydrogenase, which can provide reducing equivalents for the synthesis of PLA (Figure S3B). To further confirm the role of *P. micra* in PLA biosynthesis, we established an engineered bacteria using *E. coli* strain *BL21* to overexpresses the microbial gene *pdhD* (Figures 6A and S3C), which was significantly increased in CRC patients (Figure 3E). *BL21‐pdhD* was found to upregulate the concentration of PLA (Figure 6B). We subsequently treated NCM460 cell line with the supernatant of *BL21‐pdhD*. As expected, comet assay and immunofluorescence found that supernatant from *BL21‐pdhD* induced higher signal of DNA damage (Figure 6C,D). Transcript and protein level of γ‐H2AX also increased after stimulation with supernatant from *BL21‐pdhD* (Figures 6E,F and S3D). To confirm the role of *pdhD* in carcinogenesis, we established mice model using AOM and 2% DSS and mice were treated with engineering bacterium (Figure 6G). Compared with those mice treated with *BL21* or PBS, mice treated with *BL21‐pdhD* exhibited a significantly higher tumour number and tumour load (Figure 6H,I). These data suggested that *pdhD* gene was a key gene to mediate PLA biosynthesis, therefore promoting CRC carcinogenesis.

### 3.7 *P. micra*‐derived PLA mediates CRC carcinogenesis via AHR

But how did *P. micra*‐derived PLA mediate the DNA damage? Previous study had demonstrated that AHR was an important receptor to mediate the influence of PLA on epithelial barrier.^30^ We applied molecular docking simulation experiments and showed that PLA bound to the AHR active site such as the residue of threonine, arginine, valine and tyrosine (Figure S3E). To explore the role of AHR in PLA‐induced tumourigenesis, we constructed a AHR knockout (*Ahr^−/−^*) mice and we subsequently established a CRC tumourigenesis mice model using AOM and 2% DSS. WT and *Ahr^−/−^* mice were gavage with PLA three times a week (Figure 6J). Higher concentration of PLA was found in mice treated with PLA (Figure 6K). But we found that higher colon tumour size and tumour load in WT mice treated with PLA were significantly alleviated in *Ahr^−/−^* mice treated with PLA (Figure 6L,M). Histological assessment also confirmed the colon tumours and the intestinal DNA damage in *Ahr^−/−^* mice treated with PLA were significantly downregulated (Figures 6N and S2E). Taken together, these data demonstrated that DNA damage and CRC tumourigenesis induced by PLA were mediated by AHR signalling.

To emphasise the clinical therapeutic potential, we further evaluated the role of AHR inhibitor in *P. micra*‐induced carcinogenesis. Thus, NCM460 cell line were treated with AHR inhibitor. Significant lower transcript expression of γ‐H2AX was revealed in NCM460 cell line treated with AHR inhibitor (Figure 6O). Comet assay and immunofluorescence also found that DNA damage induced by *P. micra* and PLA was significantly alleviated in NCM460 cell line after stimulation with AHR inhibitor (Figure 6P,Q). Similar results were also found in protein level (Figures 6R and S3F,G). To confirm the therapeutic role of AHR inhibitor, we established an AOM/DSS mice model treated with AHR inhibitor as shown in Figure 6S. Consistent with the results described above, mice gavaged with *P. micra* and PLA exhibited a higher colon tumour size and tumour load (Figure 6T‒V). But AHR inhibitor could significantly alleviated the colon tumour size and tumour load in mice treated with *P. micra* and PLA (Figure 6T‒V). In addition, AHR inhibitor also downregulated the expression of γ‐H2AX in intestinal tissues (Figure S2F).

But how did AHR signalling mediate DNA damage still unclear. We found higher expression of reactive oxygen species (ROS) were detected in NCM460 cell line treated with *P. micra* and PLA (Figure S3H,I). But ROS was significantly alleviated in NCM460 treated with AHR inhibitor (Figure S3H,I). Similar trends were also observed in the expression of CYP1B1 (Figure S3J,K), which was a key intermediate molecule to mediate the effects of AHR on DNA damage. These data indicated that activation of AHR could upregulate ROS and CYP1B1, leading to the DNA damage.

## 4 DISCUSSION

In this study, we characterised the interaction of microbiota and metabolite during the development of CRC. Higher abundance of *P. micra* and higher concentration of PLA were identified in CRC patients. Our data further validated that *P. micra* was able to mediate the development of CRC via upregulation of PLA. More importantly, the role PLA in CRC was modulated by AHR and subsequently induced DNA damage in epithelium to promote tumourigenesis of CRC.

*P. micra* is an oral opportunistic pathogen, which occurs frequently in oral diseases.^31^ Recent study has been found that *P. micra* is enriched in oral squamous cell carcinoma and positively correlated with tumour metastasis. *P. micra* surface protein TmpC binds to CKAP4, a receptor overexpressed in OSCC, facilitating bacterial attachment and invasion.^32^ In recent years, it has been found that *P. micra* is not only an oral opportunistic pathogen, but also may be closely related to CRC. Many studies have reported that *P. micra* is closely involved in CRC, and can be used as a marker for CRC diagnosis.^11^ Moreover, *P. micra* has been found to be characterised by its haemolytic capacity and adherent properties, able to colonise the colonic mucosa and induce DNA methylation changes.^33^ A study also demonstrated that *P. micra* promoted the development of CRC by upregulating miR‐218‐5p expression in cells and exosomes, inhibiting *PTPRR* expression, and ultimately activating the Ras/ERK/c‐Fos signalling pathway.^34^ Consistent with current evidence, our study identified an enrichment of *P. micra* in CRC and the role of *P. micra* in tumourigenesis was further validated by different pre‐clinical models.

To evaluate the role of *P. micra* in CRC, our current study further analysed the faecal metabolites and revealed an upregulation of PLA in faeces from CRC patients. PLA from *P. micra* has also confirmed to mediate the development of CRC in mice models. Previous study had found a significantly higher levels of portal vein serum and tissue metabolites of PLA in patients with hepatocellular carcinoma, which were associated with impaired liver function and poor survival.^35^ Compared with normal control or adenoma, an enrichment of PLA was found in faecal samples from CRC patients.^28^ In addition, several genes associated with PLA metabolism was detected in the genome of *P. micra* in our current study. These results provide additional insights into the role of *P. micra* in CRC, which appears to be mediated through PLA metabolism.

Alteration of gut microbiota and metabolites are associated with intestinal homeostasis. Previous study demonstrated that aromatic lactic acids such as 3‐phenylpropionic acid from *B. fragilis* modulated intestinal epithelial barrier function via AhR signalling.^30^ Aromatic lactic acids from Bifidobacterium species were also found to modulate the CD4^+^ T cells immune response via AhR signalling.^36^ Moreover, several evidence revealed that accumulation of PLA decreased cell metabolic activity and cell viability, damaged cell membrane integrity, triggered DNA damages, and caused oxidative stress damage.^37^ Specifically, DNA damage is one of the important mechanisms underlying the occurrence of CRC.^29^ Our current data indicate that PLA from *P. micra* induces DNA damage in epithelium through AHR pathway. Targeted intervention by AHR inhibitor could alleviate epithelial DNA damage and therefore protect against the CRC development. Previous studies had demonstrated that activation of AHR could induce DNA damage via upregulation of ROS and CYP1B1. Strong oxidants in ROS can directly attack DNA molecules, resulting in base oxidation, sugar damage and DNA chain breakage.^38^ And CYP1B1 can catalyse the metabolic activation of polycyclic aromatic hydrocarbons, generating terminal carcinogens with genetic toxicity, which leads to DNA damage and gene mutations.^39^ Similar to these evidences, our results also identified that activation of AHR signalling by *P. micra* or PLA could upregulate ROS and CYP1B1, providing the potential mechanism by which *P. micra* or PLA‐induced DNA damage.

This study highlights the role of *P. micra* and PLA in CRC development. However, this work has some limitations. Although different mouse models were established in our study, the underlying mechanism how *P. micra* modulate PLA metabolism should be further explored. Several technologies such as metatranscriptomics and quantitative microbiological culture would be beneficial to validate the elevation of and *P. micra* and *pdhD* in CRC. Combination of sequencing‐based approaches with quantitative microbiological culture could improve the reliability of our conclusion. And a *pdhD*‐deficient mutant of *P. micra* would be encouraged to be established to prove the role of *pdhD* in *P. micra*‐mediated PLA biosynthesis. In addition, the impact of AHR on the epithelial DNA damage and the underlying mechanism of how PLA mediate tumourigenesis warrants further investigation.

This work uncovers the critical role of *P. micra* in CRC and demonstrates that *P. micra* was able to upregulate the PLA metabolism and subsequently induce DNA damage via AHR signalling to mediate the development of CRC. These findings present an alternative therapeutic strategy for CRC patients.

## AUTHOR CONTRIBUTIONS

Zhen He and Min Xia supervised the study and designed the experiments. Shuang Guo, Mujia Cao, Jinjie Wu, Wenhao Ma, Hongyu Xie, Yanchun Xie, Zhanhao Luo, Peng Lai, Danling Liu, Wanyi Zeng, Jingbiao Zheng, Mengze Xing, Xiqi Yin and Dayi Liang performed the experiments. Shuang Guo, Mujia Cao and Jinjie Wu collected, analysed and interpreted the data. Shuang Guo, Mujia Cao and Jinjie Wu wrote the manuscript. Jinjie Wu, Min Xia and Zhen He revised the manuscript. All the authors read and approved the final version of the manuscript.

## CONFLICT OF INTEREST STATEMENT

The authors declare they have no conflicts of interest.

## ETHICS STATEMENT

This research was conducted in line with ethical standards and received approval from Human Medical Ethics Committee of the Sixth Affiliated Hospital of Sun Yat‐Sen University (approval no. 2023ZSLYEC‐114). Written informed consent was obtained from all patients prior to sample collection.

## Supporting information



Supporting Information

## Data Availability

The metagenomic data generated in this study are publicly available at PRJNA1390667 and the whole‐genome sequencing of *P. micra* was at PRJNA1389857. The metabolomic data are publicly available at OMIX013818. Relevant raw data are available from the corresponding author upon reasonable request.
