# Supplementary material for: Parvimonas micra promotes carcinogenesis of colorectal cancer through phenyllactic acid‐induced DNA damage
Source: Clin Transl Med. 2026 May 3;16(5):e70667. doi: 10.1002/ctm2.70667 (PMC13136068; doi:10.1002/ctm2.70667)
Supplement: Supplementary file 1 — Supporting Information [file CTM2-16-e70667-s001.docx]

**Supplemental information**

**Supplemental figure title and figure legend**


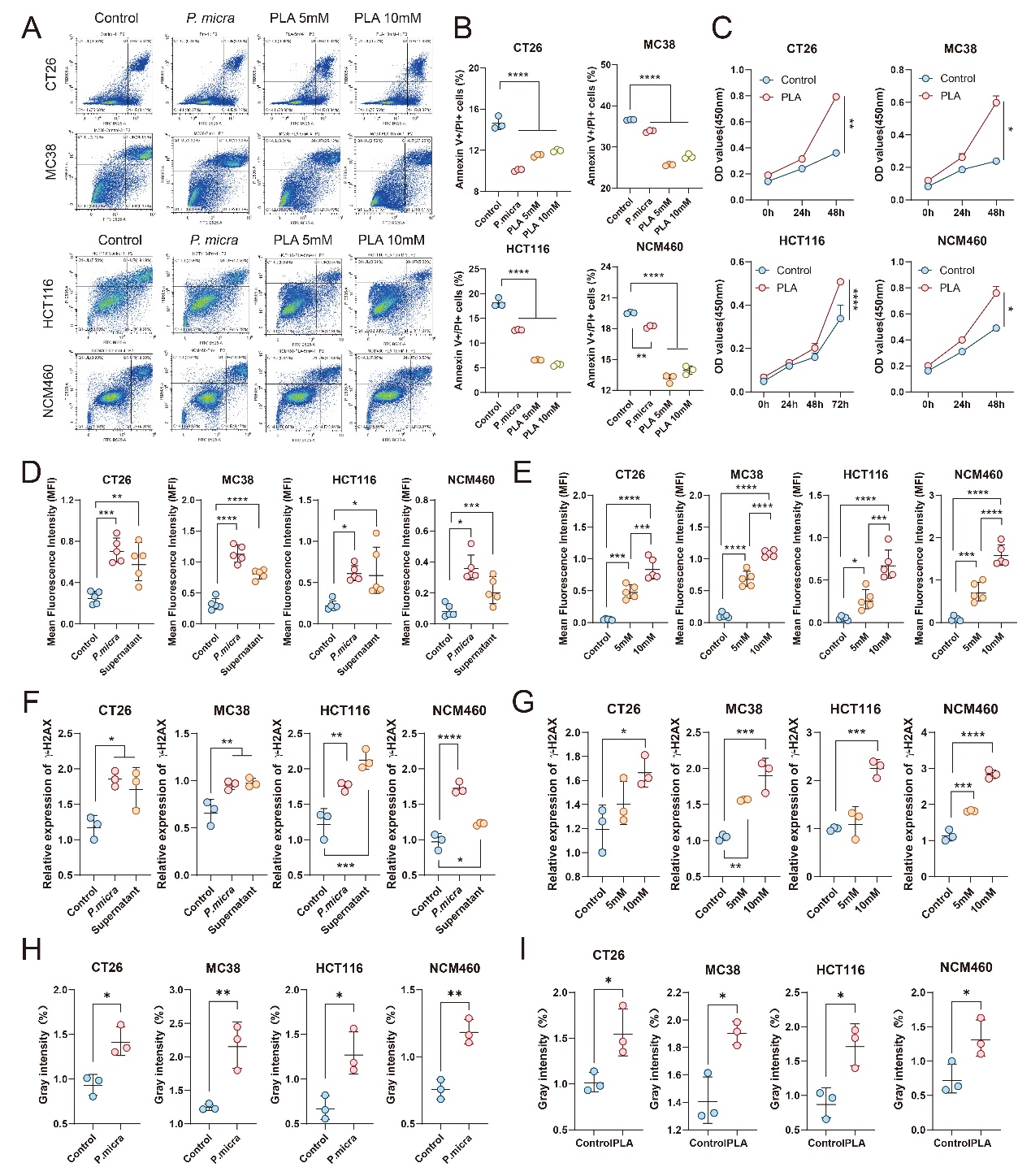


**Supplementary Figure 1. P. micra and PLA induced epithelial DNA damage. (A and B)** Apoptosis of the CT26, MC38, HCT116 and NCM460 cell line treated with P. micra or PLA. **(C)** Proliferation of the CT26, MC38, HCT116 and NCM460 cell line treated with PLA. **D)** Immunofluorescence analysis of γ-H2AX in CT26, MC38, HCT116 and NCM460 cell line treated with *P. micra*, supernatant of *P. micra* or PBS control. **(E)** Immunofluorescence analysis of γ-H2AX in CT26, MC38, HCT116 and NCM460 cell line treated with PLA or PBS control. **(F)** Transcript expression of γ-H2AX in CT26, MC38, HCT116 and NCM460 cell line treated with *P. micra*, supernatant of *P. micra* or PBS control. **(G)** Transcript expression of γ-H2AX in CT26, MC38, HCT116 and NCM460 cell line treated with PLA or PBS control. **(H)** Protein level of γ-H2AX in CT26, MC38, HCT116 and NCM460 cell line treated with *P. micra* or PBS control. **(I)** Protein level of γ-H2AX in CT26, MC38, HCT116 and NCM460 cell line treated with PLA or PBS control.


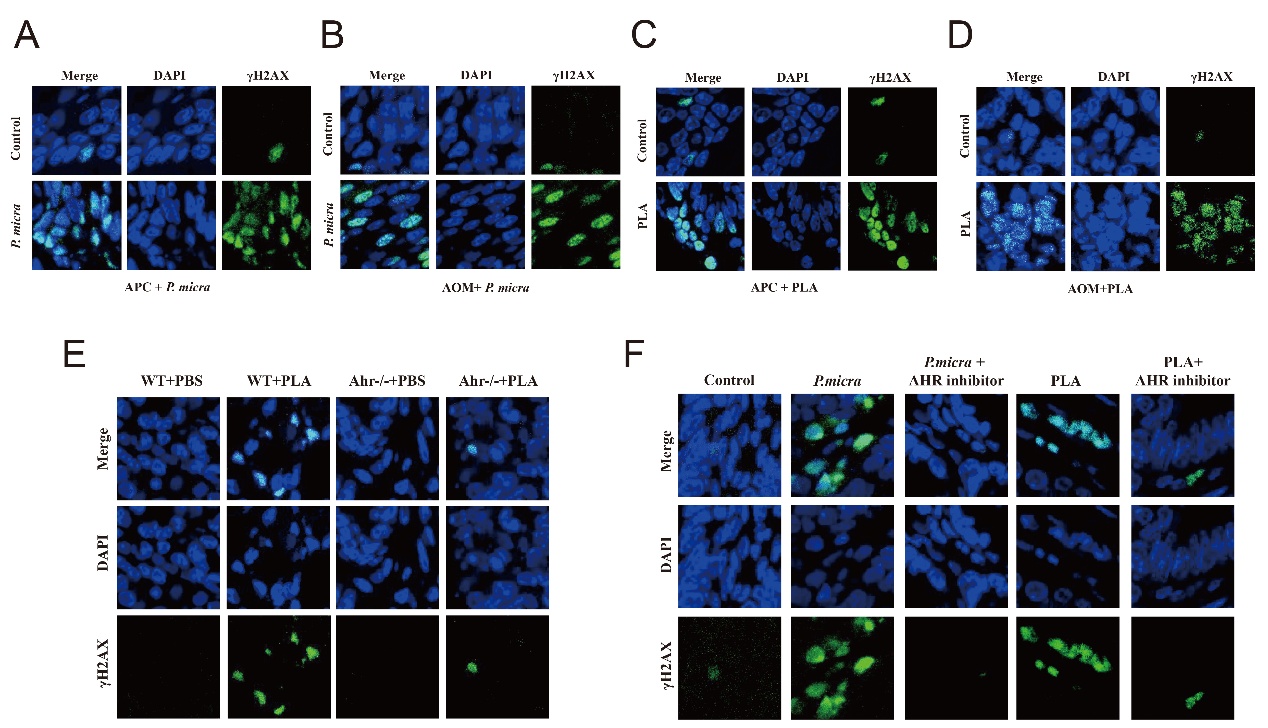


**Supplementary Figure 2. Immunofluorescence analysis of γ-H2AX in intestinal tissue from *Apc*^Min/+^ mice and AOM/DSS mice model treated with *P. micra* or PLA. (A)** Immunofluorescence analysis of γ-H2AX in intestinal tissue from *Apc*^Min/+^ mice model treated with *P. micra*. **(B)** Immunofluorescence analysis of γ-H2AX in intestinal tissue from AOM/DSS mice model treated with *P. micra*. **(C)** Immunofluorescence analysis of γ-H2AX in intestinal tissue from *Apc*^Min/+^ mice model treated with PLA. **(D)** Immunofluorescence analysis of γ-H2AX in intestinal tissue from AOM/DSS mice model treated with PLA. **(E)** Immunofluorescence analysis of γ-H2AX in intestinal tissue from WT and Ahr-/- mice model treated with PLA. **(F)** Immunofluorescence analysis of γ-H2AX in intestinal tissue from AOM/DSS mice model treated with AHR inhibitor.


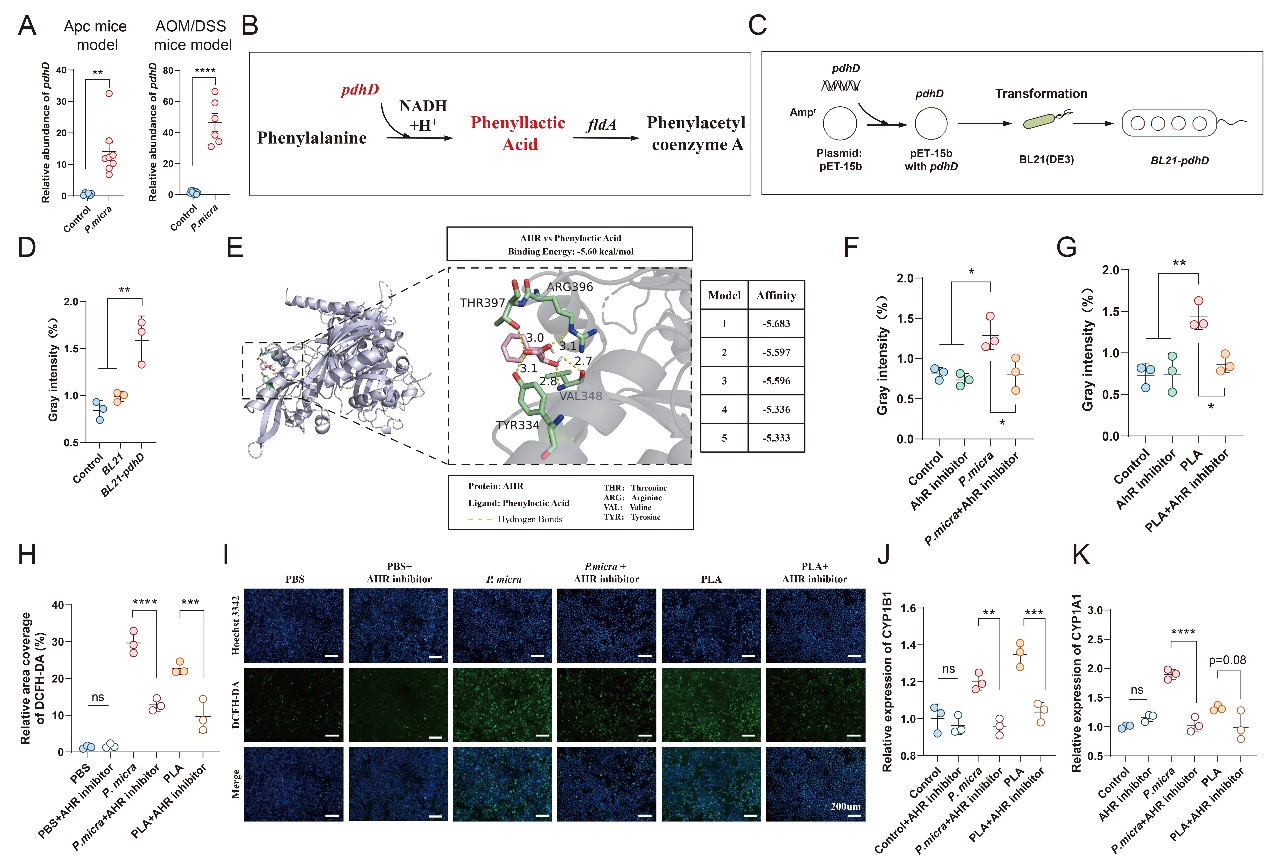
**Supplementary Figure 3. *P.micra*-derived PLA induced DNA damage via AHR signaling. (A)** Relative abundance of pdhD gene in fecal sample from *Apc*^Min/+^ mice and AOM/DSS mice model treated with *P. micra*. **(B)** Metabolic map of PLA. **(C)** Experiment workflow of the engineered bacterial construction. **(D)** Protein level of γ-H2AX in NCM460 cell line treated with supernatant from *BL21*, *BL21-pdhD* or PBS control. **(E)** Molecular docking simulation experiments between AHR and PLA. **(F and G)** Protein level of γ-H2AX in NCM460 cell line treated with AHR inhibitor. **(H and I)** Expression of ROS in NCM460 cell line treated with *P. micra* and PLA. **(J and K)** Relative expression of CYP1B1 and CYP1A1 in NCM460 cell line treated with AHR inhibitor.

**Supplementary Table 1**

|  | **Healthy control** | **CRC** | ***p* value** |
| --- | --- | --- | --- |
| n | 20 | 40 |  |
| **Sex** |  |  |  |
| female | 8(40%) | 14(35%) | 0.7797 |
| male | 12(60%) | 26(65%) |  |
| **Age (years)** | 52.35±9.07 | 58.98±9.60 | 0.0129 |
| **Height** | 1.66±0.0678 | 1.631±0.0691 | 0.2595 |
| **Weight** | 63.389±10.928 | 61.355±9.432 | 0.5726 |
| **BMI** | 22.836±2.358 | 23.050±3.188 | 0.8501 |
| **Stage** |  |  |  |
| **I** | - | 16 |  |
| **II** | - | 24 |  |
| **Histopathology** |  |  |  |
| **pMMR** | - | 39 |  |
| **dMMR** | - | 1 |  |

**Supplementary Table 1**

| Origin | Gene Name | Forward | Reverse |
| --- | --- | --- | --- |
| Human | γ-H2AX | CGGCAGTGCTGGAGTACCTCA | AGCTCCTCGTCGTTGCGGATG |
|  | CYP1B1 | GCCACTATCACTGACATCTTCGG | CACGACCTGATCCAATTCTGCC |
|  | CYP1A1 | GATTGAGCACTGTCAGGAGAAGC | ATGAGGCTCCAGGAGATAGCAG |
| Mouse | γ-H2AX | AACGACGAGGAGCTCAACAAGC | TGGCGCTGCTCTTCTTGGGCA |
| pdhD ORF | | ATGTTAACAGAAGTAATAATGCCAAAAGCTGGTAGTGAAATGGAAGAAGGCCAAATTGTAAAATGGCTTAAAAAAGAGGGAGACAAAGTTGAAGCCGGAGAAATAATTTTAGAAATTATGACTGATAAGGTTAATATGGAAATTGAAGCTGAGACTTCCGGAACTTTGCTTAAAATATTAAAACATGATGGAGAGATTGTTCCAGTAATAACAACTATTGCATATATTGGAGATGAAGGAGATGTTATACCTGAAACTGCTTCAGCACCTGTTAAAGAAGAAGTTAAGGAAGAAGTAAAAGAAAAGGTTGAAGAAAAAGTAGTTGAAGTTAAAGCTGAAACTAAAAAAGAATTAAAAGATGGTGAATATGATGTTGTTGTAATTGGTGCTGGTCCTGCAGGTTATTATTCAGCGATTAAAGCTGCTCAAAAAGGAGCAAAAGTTGCAATAGCTGAAAATAATAAATTTGGTGGAACTTGCTTAAATAGAGGATGTATTCCAACAAAAACATATTTACAAAATGTTGAAGATTTGGAAAGAATTAAAGCATCAAGTAAAAGAGGAATTATTTTAGAAAATGATAAAGCTACTGTTGATGTTTCAAAGGCTTTAAAATTTAAAAATTCAATCGTAAAGAAACTTACTGCTGGTGTTGAATTCTTATTAAAGAGCAATTCAGTAGAAATGTTTAAAGAAACAGCTTATATTAATTCAAATGGAAATGTTACTTTAGAAAGTGGAAAAGAACTTGTATGTGGTTCTGTGATTTTTGCCGGTGGTTCTAAATGTGTTAAAAATATCAAAGGATCAGATAGCTCAAATGTTATAGATACTGATGAAGCTTTAGATTTAAAAGAAGCACCTGAATCATTAGTAATAATCGGAGCAGATTATATTGGAGTTGAAATGGCTCAAATATTCAGTTCTTTCGGAAGCAAAGTGACTGTTGTTGAAAGAAAAGATTCAGCGGTTGAAGTTATAGATTCAGAAGTTTCTTCAATTTTAATAAAGTCTTTAGAAAAATCAGGAATTAAATTTATCTTTGGTAAAGAAATTACAGAAATTTCCGGAGAAAAAGTATTAGCCGGTTCAGAAGAAGTTGCAAGTGCAAAAGTAATTTTACTTACAACTAGAGAAGCTGATTTAACAGCATTAAAAGATGTAAATCTTGAAGTTTCAAATGGAAATGTAGTTGCTAATGAAAAAATGCAATCAAGTTTAAAGAATATTTATGTTCCTGGAGATGTTAATGGTAAAAATCTTCTTGCACATGCTGCATTTAAAATGGGATATGTTGCTGCTTCAGAAATAGTTGAAGGTAAATCAGACAAATACAATAACAATATTATTCCAAGAGCAATTTACACATATCCTGAAATAGGAAGTGTAGGTTTAACAGAAGAAGAAGCAAAGAAATCATATGATGTTAAAGTTGGAAAATTCAATTATGGAGCAAATGGAAGAGCTTTAGCCCATGGAGATTCATCAGGAATGGTTAAAATCATTTCAGATGCAAGATATGGAGAAATCTTAGGAGCTCATATTGTTGGACCTAGAGCATCCGAGTTGATTAATGAAGTTTCAATTTTAATGCAATCAGAAGTTATTGTTGAAGAAGCAATTAAAATGGTATTTGGACATCCAACTTTCTCAGAAGCAATTTATGAAGCAATTGCTGATGTAGAAGGAGTTAGTGTACATTTGCCTAAAAAATAA | |
